# MicroRNA-223-3p Inhibits the Angiogenesis of Ischemic Cardiac Microvascular Endothelial Cells via Affecting RPS6KB1/hif-1a Signal Pathway

**DOI:** 10.1371/journal.pone.0108468

**Published:** 2014-10-14

**Authors:** Guo-Hua Dai, Pei-Ze Ma, Xian-Bo Song, Ning Liu, Tong Zhang, Bo Wu

**Affiliations:** 1 Affiliated Hospital of Shandong University of Traditional Chinese Medicine, Jinan, China; 2 Shandong University of Traditional Chinese Medicine, Jinan, China; University Hospital Medical Centre, Germany

## Abstract

**Background:**

MicroRNAs (miRNAs) are a recently discovered class of posttranscriptional regulators of gene expression with critical functions in the angiogenesis and cardiovascular diseases; however, the details of miRNAs regulating mechanism of angiogenesis of ischemic cardiac microvascular endothelial cells (CMECs) are not yet reported.

**Methods and Results:**

This study analyzes the changes of the dynamic expression of miRNAs during the process of angiogenesis of ischemic CMECs by applying miRNA chip and real-time PCR for the first time. Compared with normal CMECs, ischemic CMECs have a specific miRNAs expression profile, in which mir-223-3p has the most significant up-regulation, especially during the process of migration and proliferation, while the up-regulation is the most significant during migration, reaching 11.02 times. Rps6kb1 is identified as a potential direct and functional target of mir-223-3p by applying bioinformatic prediction, real-time PCR and Western blot. Pathway analysis report indicates Rps6kb1 regulates the angiogenesis by participating into hif-1a signal pathway. Further analysis reveals that both the gene and protein expression of the downstream molecules VEGF, MAPK, PI3K and Akt of Rps6kb1/hif-1a signal pathway decrease significantly during the process of migration and proliferation in the ischemic CMECs. Therefore, it is confirmed that mir-223-3p inhibits the angiogenesis of CMECs, at least partly, via intervening RPS6KB1/hif-1a signal pathway and affecting the process of migration and proliferation.

**Conclusion:**

This study elucidates the miRNA regulating law in the angiogenesis of CMECs; mir-223-3p inhibits the process of migration and proliferation of ischemic CMECs probably via affecting RPS6KB1/hif-1a signal pathway, which in turn suppresses the angiogenesis. It is highly possible that mir-223-3p becomes a novel intervention core target in the treatment of angiogenesis of ischemic heart diseases.

## Introduction

Myocardial ischemia and myocardial infarction are the most common and frequently encountered diseases in clinic. Present studies haven't revealed the function and mechanism of endogenous factor in ischemia myocardial. MicroRNAs are a class of recently-discovered endogenous non-coding RNAs (∼22 nt), which pair-bond 3′ non-coding region of target gene mRNAs and negatively regulate the expression of target mRNAs post-transcriptionally, in addition to playing a vital role in the cell differentiation, proliferation, apoptosis, individual development and the body metabolism [1]–[4]. According to the bioinformatics analysis, one miRNA may have hundreds of potential target mRNAs and about 30% of human genes are controlled by the miRNA, which indicates miRNA probably affects the entire signal pathway [5], [6]. MiRNA has tissue specificity and time sequence and it expresses on the specific tissues and during the specific development stage, and in this way, it regulates the expression of target genes dynamically [7].

When myocardial ischemia attacks, it is highly possible that the dynamic change of miRNA affects the expression of lots of target protein, which in turn damages the construction and function of the ischemic myocardium. Therefore, to study miRNA that is closely related to the angiogenesis will contribute to the treatment of ischemic diseases by applying newly-discovered ways of regulating angiogenesis. From analyzing the expression of the human umbilical vein endothelial cell (HUVEC) miRNA, Fish, etc. find that miRNA-126 is a specific endothelial cell miRNA out of 15 high-expressed miRNAs, which promotes angiogenesis and the formation of vascular integrity [8]. While Poliseno, etc. identify 27 high-expressed miRNAs in HUVEC, and confirm that miR-221/222 inhibits the angiogenesis via regulating the expression of the C-KIT gene [9]. Currently, there are incongruous reports on the miRNA target related to the angiogenesis, which vary from miR-126 [10], miR-130a [11], miR-378 [12], and miR-27b [13] to miR-210 [14], all of which are regarded as miRNAs which promote the angiogenesis while miR-92a [15], miR-221 [16] and miR-222 inhibit the migration and proliferation of endothelial cells and the angiogenesis. However, further experiments are needed to confirm which one is at the core or main position, whether it is a kind of miRNA or a group of miRNAs, which will become the therapeutic target in the treatment of ischemic diseases. Only a small proportion of the dynamic expression of miRNA is revealed, thus the discovery of the regulating expression of miRNA during the pathophysiologic process of the vascular diseases and the target genes will contribute to a keener understanding of the function miRNA in the vessels.

When the ischemic vascular diseases attack, the compensation of endogenous collateral circulation in the body is limited, namely, the angiogenesis of endothelial cells is restricted. However, the feature of the angiogenesis of the ischemic CMECs has not been reported yet.

Based on the above mentioned knowledge, this study analyzes the changes of the dynamic expression of miRNAs during the process of angiogenesis of CMECs by applying miRNA chip and real-time PCR for the first time and confirms the key miRNA in three different stages of the ischemic CMECs proliferation, migration and tube formation respectively. It is found in this study that the expression of mir-223-3p in the angiogenesis of the ischemic CMECs increases significantly and Rps6kbl is the target gene of mir-223-3p, it is also revealed that mir-223-3p inhibits the process of migration and proliferation of ischemic CMECs via affecting RPS6KB1/hif-1a signal pathway, which finally suppresses the angiogenesis.

## Materials and Methods

### Rats myocardial infarction model building and cell culture

The study was approved by the Ethic Committee for the Protection of Animal Care of Shandong University of TCM (Jinan, China). 38 male SD rats (220∼280 g) were purchased from Shandong University of TCM Experimental Animal Center (Jinan, China). The rats myocardial infarction models were made as previously described by Drexler H et al. [17]: rats were anesthetized by 10% chloral hydrate, 0.3 mL/100 g and their electrocardiograms were recorded after being fixed, cervical trachea was isolated, breathing machine was connected after the chest was opened and the heart was exposed, left anterior descending coronary artery (LAD) was ligated, ST segment elevation, T wave ascending or inverting in the recorded electrocardiograms proved the ligation was successful. Rats woke up from anesthesia in 1–3 hours after operation. Those survived 24 hs after the operation became models. General characteristics of myocardial infarcted rats, such as body weight, mental condition, respiratory frequency, animal hair, urine, feces and etc., were monitored twice a day before ischemic CMECs were isolated from the apex of myocardial infarction model rats for about 2weeks. The food and water consumption, animal activity, expression in eyes, color of the tails and etc. of myocardial infarction model rats were observed after surgery. The temperature, humidity and ventilation of the feeding chamber were regulated timely. Providing the sterilized water and changing the bedding for the rats everyday to ensure animal welfare. Meanwhile, weak rats or rats before recovery after anesthesia were kept separately to prevent them from being attacked. Ischemic CMECs were isolated from the apex of myocardial infarction model rats and cultured using previously reported methods [18] and normal CMECs were isolated from the apex of healthy male SD rats, also cultured as described [18]: rats were anesthetized by 10% chloral hydrate, 0.3 mL/100 g and were sterilized by immersing in 75% ethanol for 8 minutes, then their chests were opened and their hearts were sheared under sterility, and rats were euthanized by overdose anesthesia with 10% chloral hydrate (0.6 mL/100 g) after hearts harvest in the experiment. The hearts were next put in sterile culture medium, their great vessels, both of the atrium cordis, ventriculus dexter and interventricular septum were dislodged, then they were rinsed in PBS repeatedly, the rest of the ventricular muscle was transferred into a beaker and was cut into pieces of 2 mm^3^ by using ophthalmic scissors, then, they were inoculated evenly into a culture flask with 1 ml fetal bovine serum and cultured statically for 4 hs at 37°C in 5% CO_2_ in a humidified incubator, which was followed a continuous culture of 60∼70 h in added 2 ml DMEM high glucose medium (Gibco, Carlsbad, CA) containing 20% fetal bovine serum (FBS; Gibco), tissue masses were removed and DMEM high glucose medium was changed per 3 days till the cells reached fusion. VIII factors and CD31 were identified by immunocytochemistry. All the rats involved were euthanized by overdose anesthesia with sodium pentobarbital (80 mg/kg, i.p.) after the experiment.

### Cell proliferation

Cell proliferation was detected by using MTT methods as previously described [19]–[22]. Briefly, the normal CMECs and ischemic CMECs were resuspended in 10% fetal calf serum medium respectively, and then made into single cell suspension, which was inoculated into sixteen 96-well cell culture plates, 4000 cells per well, 8 plates for one group, 200 µ for one well, and 6 duplicate well for every group. 20 µL of a 5 g/L MTT solution was added to each well ten hours, one day, two days, three days, four days, five days, six days, seven days after inoculation respectively and culture was terminated after another incubation of 4 hs at 37°C. Next, the medium was carefully aspirated from each well and 150 µL DMSO was added, absorbance was detected at a wavelength of 490 nm by using microplate reader (Bio-Tek, ELX 800, USA) and the proliferation rate was calculated to find the window phase of proliferation, and the cell growth curve was finally drawn with time as abscissa and observance as ordinate.

### Cell migration

Scratch test was applied to detect the migration of cells [23]. When the cell growth reached 80% fusion, two groups of cells digestion were inoculated into 24 well plates, six duplicate wells for each group, a cross was scratched in the middle of the bottom of 24 well plates with steriled 10 µL spear, cultured at 37°C in 5% CO_2_ in an incubator and took a picture of the intersection of the scratch zero hour, one day, two days, three days, four days respectively after the scratch to observe the healing power, to count the number of cell migration, to calculate the migration rate and to find the window phase of migration.

### Tube formation

CMECs tube formation was observed by using inverted phase contrast microscope and the formation was counted. When the cell growth reached 80% fusion, two groups of cells digestion were inoculated into two 6 well plates, six duplicate wells for each group, tube formation was observed by using inverted phase contrast microscope (100×) one day, two days, three days, four days respectively after the inoculation, a tube was formed when the connection of endothelial cell showed “C” [24]–[25]. The number of the tube formation was counted in the amplificated (100×) vision from three most intense tube gathering vision per well. 10 pictures of each group were taken randomly to get the average in order to calculate the tube formation rate to find the window phase of tube formation.

### MiRNA gene chip analysis

Based on the “window” feature of normal/ischemic CMECs angiogenesis process, two groups of cells were further divided according to the time of proliferation, migration and tube formation. The total RNA was extracted from the cells of each group (the total number is about 2×10^6^). RNA quality was detected by measuring OD260/OD280 ratio and using formaldehyde denaturation agarose gel electrophoresis, the ratio varied from 1.8 to 2.1, which indicated that the quality of RNA was reliable. MiRNA isolation, fluorescence labeling, microarray hybridization experiments and data analysis were made according to manufacturer's instructions. MiRNA was labeled by using miRCURY Array Power Labeling kit. The labeled sample was concentrated by using Rneasy mini kit. MiRNA microarray hybridization was made by using miRCURY Array microarray kit and Hybridization Chamber II kit. Fluorescence excitation was made at 635 nm and the Axon GenePix 4000B microarray scanner (Axon Instruments, Foster City, CA) was used to scan the images, scanned images were then imported into GenePix Pro 6.0 software (Axon) for the digital transformation of the microarray images, the median normalization method was used to calculate the standard value. Firstly, differential expression ≥2times is used as criterion, according to the gene expression, differential expressed miRNA was filtered and given a cluster analysis, which included miRNA that had a more than 2 times up-regulated-expression and miRNA that had a less than 2 times down-regulated-expression, the key of which was to choose miRNA with a significant differential expression. Secondly, major miRNA related to proliferation, miRNA related to migration and miRNA related to tube formation were spotted according to the code of differential expression during stages of miRNA proliferation, migration and tube formation, the core miRNA of ischemic CMECs was worked out. Microarray hybridization experiments and consequent data analysis were finished by Shanghai KangCheng Bio-technology.

### Real-time PCR testing and verifying core miRNA

As is described in the previous part, two groups of cells were further divided according to the time of proliferation, migration and tube formation to detect the expression of core miRNA in different groups. The total RNA was extracted from the cells of each group and the purity and density of RNA were detected. RNA sample primer was given a reverse transcription into cDNAs according to reverse transcriptase. All cDNA samples were allocated to real-time PCR reaction system respectively, which followed the following procedure: Reactions were incubated at 95°C for 10 minutes, followed by 40 PCR cycles of 95°C for 10 seconds, 60°C for 60 seconds to get the primary curve; after the amplification reaction, reactions were incubated at 95°C for 10 seconds, 60°C for 60 seconds and 95°C for 15 seconds; the temperature increased from 60°C to 99°C gradually (the test system carried it out automatically at a Ramp Rate of 0.05°C/second) to establish the solubility curve of PCR primer. The data were analyzed by using 2^−ΔΔCt^ method.

### MiRNA target prediction

MiRNA target gene wasere predicted by retrieving data bases of mirbase (http://www.ebi.ac.uk/, mirbase(http://www.ebi.ac.uk/) and mirdb (http://mirdb.org/miRDB/), a predictive analysis of the secondary structure and target gene of miRNA was made to spot the gene with a characteristic of a high degree of sequence matching, stable secondary structure and target sequences being highly conserved among species. The gene predicted in this three software simultaneously was given a GO analysis and pathway analysis to spot the target gene, which was closely related to angiogenesis.

### Reverse-transcription and real-time Polymerase Chain Reaction (PCR)

As is described in the previous part, two groups of cells were further divided according to the time of proliferation, migration and tube formation, the miRNA expression of Rps6kb1, HIF-1a, VEGF, MAPK, PI3K and Akt was detected by applying real-time PCR.

### Western blot analysis

As is described in the previous part, two groups of cells were further divided according to the time of proliferation, migration and tube formation, the gene expression of Rps6kb1, HIF-1a, VEGF, MAPK, PI3K and Akt was detected by applying Western-blot [26]. Total protein extraction kit was used to extract the total protein of the samples. The density of the samples was measured according to the instruction of BCA protein fluorometric kit. The protein was ionophortically separated after the prepared sample and prestained protein marker were loaded respectively. The filter paper cellulose gel interlayer was assembled according to the instruction of Bio-Rad protein transferring device. Non specific binding on the closing membrane was incubated at room temperature in 5% skim milk powder solution. First antibody was added into sealed membrane to achieve the antigen antibody union. HRP labeled second antibody was added to unite first antibody. HRP labeled β-actin antibody was added to detect the content of β-actin simultaneously. The membrane was incubated with chemiluminescence substrate and developed after X film exposure. The pictures were scanned and GIS1000 Software was applied to digitalize the grey level of every specific band of the pictures. The grey level of the target protein was divided by that of β-actin to get alignment error, of which the outcome stood for the relative content of the target protein of the specific sample.

### Statistical analysis

All data are expressed as mean±standard deviation (SD) from at least three separate experiments. The differences between groups were analyzed using Student's *t* test. Differences were of statistical significance when *P*<0.05.

## Results

### Rats myocardial infarction model building and cell culture

**Figure 1:**
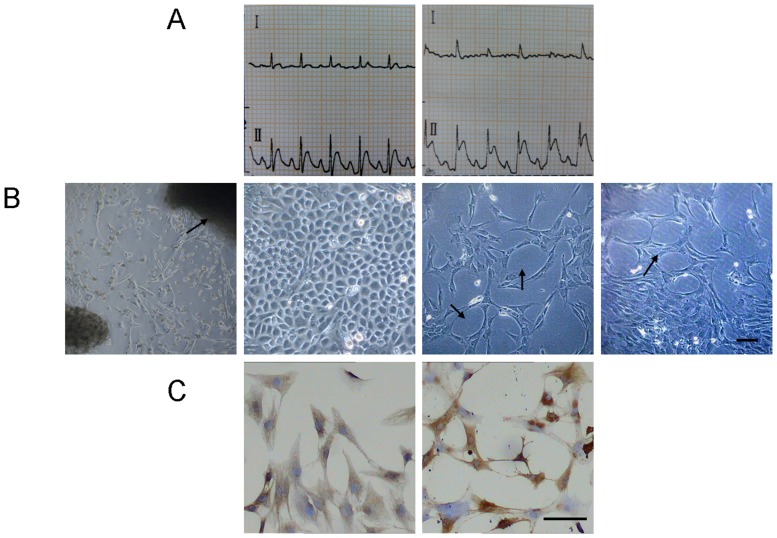
A. Left: a normal electrocardiogram or rat before the model building. Right: After the model building, electrocardiograms lead II indicated ST segment elevated significantly, which indicated ischemia myocardial and proved the artery ligation was successful. B. The cells were in the shape of stars or polygons when they initially disassociated out of the tissue block and had a low density, they were in the shape of paving stones when the cells bespreaded the bottom of the culture flask, in some of which tube structure and vessel network structure could be spotted. C. Immunocytochemical stain revealed that: (left) after the staining of VIII factor, the cytoplasm was brown coloring, the coloring was the most significant in pericaryon; (right) after the staining of CD31, their cell membrane showed yellowish brown particles, which proved that the cultured cells were CMECs.

After the rat's left anterior descending coronary artery (LAD) was ligated, electrocardiograms indicated ST segment elevated significantly (Fig. 1A), which proved the model building was successful. There were two rats failed in respiration and circulation in 24 hours after mice model establishment, and breathing machine and intravenous injection of 0.1% adrenaline 1 mL were given, and finally these two rats died despite the rescuing efforts. Finally 38 of the myocardial infarction model rats were established and 36 were survived (success rate was 94.7%). Under reverted microscope, cells were observed to disassociate out of the tissue block and bespread the bottom of the culture flask, during which typical morphological and functional changes with typical CMECs characteristics (Fig. 1B) were found. Immunocytochemical stain revealed that VIII factor and CD31 expressed positively in cells (Fig. 1C), which proved that the cultured cells were CMECs.

### Cell growth curve and proliferation

**Figure 2:**
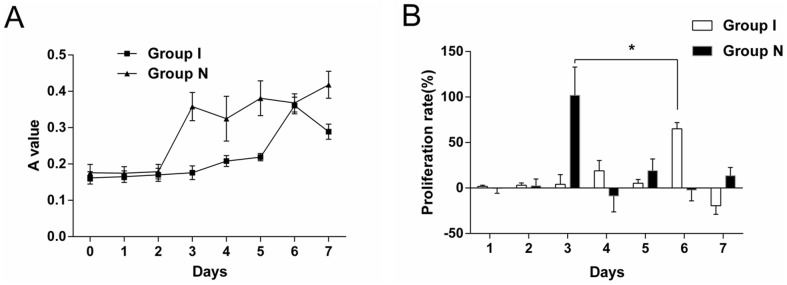
A. The OD value of normal CMECs exceeded that of ischemic CMECs, the normal CMECs proliferated vigorously on the third day while the cell growth curve of ischemic CMECs was even, the OD value reached a peak till the sixth day. B. A dynamic observation revealed that the proliferating phase of the normal CMECs was the third day while the proliferating phase of the ischemic CMECs was the sixth day. The cell proliferation ratio of ischemic CMECs decreased significantly compared with that of the normal CMECs (P<0.05).

MTT colorimetry method was used to protract the cell growth curve, the normal CMECs grew slowly during the first 2 days, proliferated vigorously on the third day at a logarithmic growth, and then went into a cell growth plateau. Ischemic CMECs grew slowly during the first 5 days, and proliferated vigorously on the sixth day, during which there was no obvious plateau (Fig. 2A). The window phases of cell proliferation of both groups were found respectively from the dynamic observation (Table 1). The cell proliferation ratio of ischemic CMECs decreased significantly compared with that of the normal CMECs (*P*<0.05) (Fig. 2B).

**Table 1:** The window phases of proliferation, migration, tube formation of the two groups.

Group	n	migration	tube formation	proliferation
ischemia	6	The first day	The second day	The sixth day
normal	6	The first day	The second day	The third day

### Cell migration and tube formation

**Figure 3:**
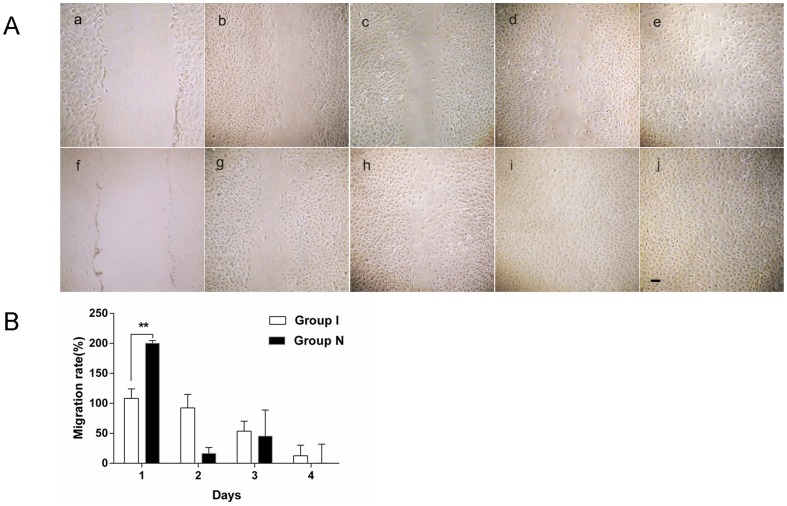
A. Under microscope, for normal CMECs, a clear scratching blank area could be spotted after the scratching test. The blank area decreased significantly with amount of cells crossed the scratching line one day later. The blank area was gradually covered with increasing proliferated cells two days later. The blank area was completely covered with proliferated cells three days later. For ischemic CMECs, a similar scratching blank area could also be spotted after the scratching test. The blank area could still be clearly spotted one day later because there was only a small amount of cell migrated to the blank area. The migrated cells increased two days later, but were still less than that of normal CMECs. The migrated cells increased three days later, but the blank area could still be spotted. B. A dynamic observation revealed that the cell migration ratio of ischemic CMECs decreased significantly compared with that of the normal CMECs (*P*<0.01).

**Figure 4:**
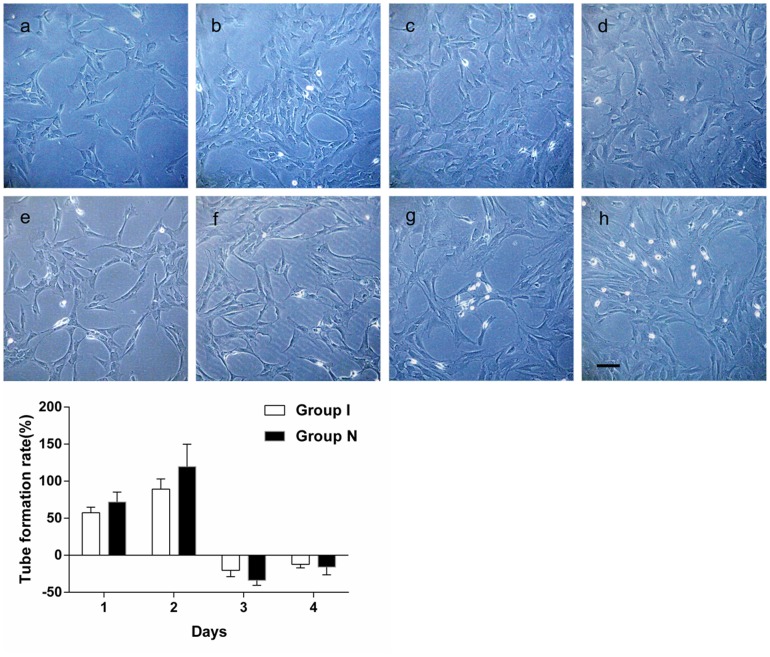
A. For normal CMECs, observation under inverted phase contrast microscope (100×) revealed that amounts of “C”-shaped tube structure were formed one day after inoculation. The “C”-shaped tube structures were clearer and increased significantly two days after inoculation. The “C”-shaped tube structure decreased with an increased cell number three days after inoculation. The “C”-shaped tube structure further decreased four days after inoculation. For ischemic CMECs, there was no obvious tube formation one day after inoculation, only several “C”-shaped structure were spotted under inverted phase contrast microscope (100×). Several tube formations were spotted two days after inoculation, of which the number was less than that of normal CMECs. The tube structure decreased with an increased cell number three days after inoculation. The tube structure disappeared four days after inoculation. B. A dynamic observation revealed that the migration phase of normal/ischemic CMECs was the second day. Tube formation ratio of ischemic CMECs decreased compared with that of the normal CMECs, but that was of no statistical significance.

The number of cell migration of ischemic CMECS decreased significantly compared with that of the normal CMECs (Fig. 3A). The window phases of cell migration of both groups were found respectively from the dynamic observation (Table 1). The cell migration ratio of ischemic CMECs decreased significantly compared with that of the normal CMECs (*P*<0.01)(Fig. 3B). The number of tube formation of ischemic CMECS decreased significantly compared with that of the normal CMECs (Fig. 4A). The window phases of cell tube formation of both groups were found respectively from the dynamic observation (Table 1). The tube formation ratio of ischemic CMECs decreased compared with that of the normal CMECs, but that was of no statistical significance (*P*>0.05) (Fig. 4B).

### The differential expression of miRNAs during the angiogenesis of ischemic CMECs

**Figure 5:**
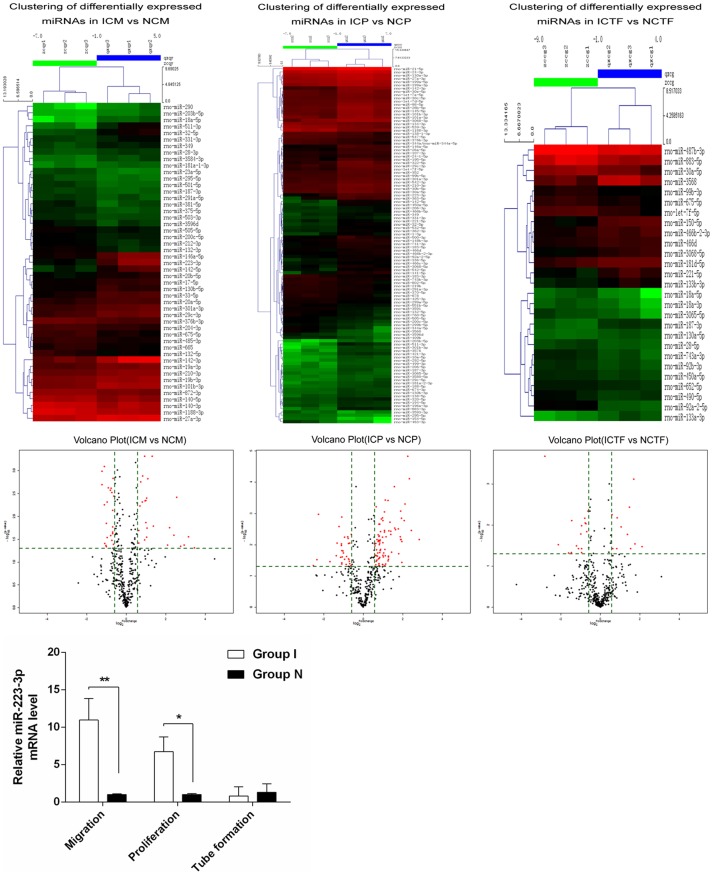
MicroRNA expression profiling in ischemic CMECs proliferation(ICP), ischemic CMECs tube formation (ICTF)and ischemic CMECs migration (ICM)compared with that of normal CMECs proliferation(NCP), normal CMECs tube formation (NCTF) and normal CMECs migration (NCM). A. Differentially expressed miRNAs in ICP(n = 3), ICTF(n = 3) and ICM(n = 3) compared with NCP(n = 3), NCTF(n = 3) and NCM(n = 3). The heat map diagram showed the result of the two-way hierarchical clustering of miRNAs and samples. Each row represented a miRNA and each column representsed a sample. The miRNA clustering tree was shown on the left, and the sample clustering tree appeared at the top. The color scale shown at the top illustrated the relative expression level of a miRNA in the certain slide: red color represented a high relative expression level; green color represented a low relative expression levels. n = 3 in each group. B. Volcano Plots were useful tool for visualizing differential expression between two different conditions. They were constructed using fold-change values and p-values, and thus allowing visualizing the relationship between fold-change (magnitude of change) and statistical significance (which took both magnitude of change and variability into consideration). They also allowed subsets of genes to be isolated, based on those values. The vertical lines corresponded to 1.5-fold up and down, respectively, and the horizontal line represented a p-value of 0.05. So the red point in the plot represented the differentially expressed miRNAs with statistical significance. C. Confirmation of mir-223-3p in the same set of samples as used in microarray assay by means of real-time PCR. ΔCt values were normalized to U6 levels. Relative expression was calculated with respect to normal CMECs. The results were expressed as Log10 (2∧^−ΔΔCt^). *P<0.05, **P<0.01.

At present, the expression and function of miRNAs in ischemic CMECs at different stages of angiogenesis is largely unknown. This study explored the expression profile of miRNAs during the different stages of angiogenesis of CMECs by applying miRNAs chip for the first time. Based on the “window” feature of normal/ischemic CMECs angiogenesis process, two groups of cells were further divided according to the time of proliferation, migration and tube formation. Compared with normal CMECs, ischemic CMECs had 40 up-regulated miRNAs and 16 down-regulated miRNAs during proliferation, normal CMECs proliferation (NCP), ischemic CMECs proliferation(ICP) (Table 2), ischemic CMECs had 7 up-regulated miRNAs and 7 down-regulated miRNAs during tube formation, normal CMECs tube formation (NCTF), ischemic CMECs tube formation (ICTF) (Table 3), and ischemic CMECs had 16 up-regulated miRNAs and 6 down-regulated miRNAs during migration, normal CMECs migration (NCM), ischemic CMECs migration (ICM) (Table 4). According to the significance of differential expression and its relationship with angiogenesis, mir-142-5p was confirmed as the key miRNA of the ischemic CMECs proliferation; mir-223-3p was confirmed as the key miRNA of the ischemic CMECs migration; mir-221-5p was confirmed as the key miRNA of the ischemic CMECs tube formation (Table 5). Mir-223-3p had the most significant up-regulation during angiogenesis of ischemic CMECs, in which it increased 3.679 times during proliferation and 11.022 times during migration. These data indicated that up-regulated mir-223-3p played an important role in the angiogenesis of ischemia CMECs. Finally mir-223-3p was confirmed as the core miRNA of ischemic CMECs. Expression heat maps of the data were presented in Figure 5A. Volcano Plots were presented in Figure 5B.

**Table 2:** The differential expression of miRNAs in ICP and in NCP (n = 3 in each group).

Gene	Fold change	*P* value
**Up-regulated miRNA in ICP**		
rno-miR-511-3p	7.157	0.007
rno-miR-301b-3p	5.408	0.004
rno-miR-142-5p	5.022	0.00007
rno-miR-142-3p	4.776	0.00001
rno-miR-421-3p	4.346	0.0005
rno-miR-3576	4.213	0.002
rno-miR-203b-5p	3.858	0.037
rno-miR-363-5p	3.855	0.008
rno-miR-10a-5p	3.843	0.001
rno-miR-450a-5p	3.757	0.007
rno-miR-141-5p	3.721	0.007
rno-miR-883-3p	3.711	0.001
rno-miR-223-3p	3.679	0.006
rno-miR-322-5p	3.494	0.019
rno-miR-146a-5p	3.409	0.003
rno-miR-292-5p	3.096	0.003
rno-miR-29c-3p	3.015	0.003
rno-let-7f-5p	2.976	0.0001
rno-miR-26b-5p	2.709	0.018
rno-miR-499-3p	2.652	0.004
rno-miR-32-5p	2.647	0.005
rno-miR-98-5p	2.573	0.002
rno-miR-206-5p	2.453	0.004
rno-miR-145-5p	2.437	0.029
rno-miR-101b-3p	2.409	0.023
rno-miR-187-3p	2.408	0.025
rno-miR-195-5p	2.384	0.033
rno-miR-362-3p	2.381	0.0004
rno-miR-336-5p	2.246	0.006
rno-miR-1-3p	2.193	0.042
rno-miR-30e-5p	2.181	0.023
rno-miR-532-5p	2.165	0.0004
rno-miR-29c-5p	2.149	0.014
rno-miR-26a-5p	2.132	0.048
rno-miR-741-3p	2.123	0.001
rno-miR-210-3p	2.122	0.004
rno-miR-188-5p	2.112	0.003
rno-miR-181a-2-3p	2.058	0.018
rno-miR-3068-3p	2.025	0.025
rno-let-7d-5p	2.003	0.002
**Down-regulated miRNAs in ICP**		
rno-miR-463-3p	0.175	0.048
rno-miR-3560	0.197	0.031
rno-miR-154-5p	0.208	0.001
rno-miR-344a-5p	0.312	0.035
rno-miR-3596d	0.393	0.023
rno-miR-1188-3p	0.402	0.009
rno-miR-295-5p	0.429	0.003
rno-miR-760-5p	0.454	0.026
rno-miR-539-3p	0.468	0.009
rno-miR-409b	0.468	0.011
rno-miR-299b-5p	0.472	0.016
rno-miR-183-3p	0.474	0.039
rno-miR-3593-3p	0.474	0.043
rno-miR-3591	0.477	0.001
rno-miR-878	0.479	0.025
rno-miR-200c-5p	0.494	0.016

**Table 3:** The differential expression of miRNAs in ICTF and in NCTF (n = 3 in each group).

Gene	Fold change	*P* value
**Up-regulated miRNA in ICTF**		
rno-miR-133a-3p	4.334	0.034
rno-miR-3568	3.401	0.029
rno-miR-150-5p	3.233	0.001
rno-miR-221-5p	2.919	0.037
rno-miR-187-3p	2.802	0.004
rno-miR-133b-3p	2.586	0.017
rno-miR-3068-5p	2.073	0.011
**Down-regulated miRNAs in ICTF**		
rno-miR-92a-2-5p	0.144	0.0002
rno-miR-18a-5p	0.230	0.029
rno-miR-3065-5p	0.329	0.047
rno-miR-18a-3p	0.362	0.046
rno-miR-743a-3p	0.369	0.007
rno-miR-490-5p	0.437	0.032
rno-miR-883-5p	0.467	0.037

**Table 4:** The differential expression of miRNAs in ICM and in NCM (n = 3 in each group).

Gene	Fold change	*P* value
**Up-regulated miRNA in ICM**		
rno-miR-223-3p	11.022	0.049
rno-miR-511-3p	8.907	0.028
rno-miR-142-3p	7.591	0.042
rno-miR-142-5p	6.739	0.045
rno-miR-18a-5p	5.868	0.003
rno-miR-146a-5p	5.326	0.026
rno-miR-32-5p	4.581	0.017
rno-miR-290	4.287	0.042
rno-miR-203b-5p	2.597	0.016
rno-miR-33-5p	2.503	0.0004
rno-miR-101b-3p	2.138	0.001
rno-miR-3584-3p	2.095	0.018
rno-miR-29c-3p	2.055	0.004
rno-miR-331-3p	2.054	0.028
rno-miR-349	2.010	0.004
rno-miR-20b-5p	2.008	0.013
**Down-regulated miRNAs in ICM**		
rno-miR-132-3p	0.428	0.002
rno-miR-23a-5p	0.428	0.001
rno-miR-375-5p	0.465	0.001
rno-miR-501-5p	0.468	0.005
rno-miR-381-5p	0.475	0.039
rno-miR-132-5p	0.495	0.043

**Table 5:** Results of miRNA gene chip analysis.

Group	Gene name	Fold change	Regulation
ICM	mir-223-3p	11.02	up
ICP	mir-142-5p	5.02	up
ICTF	mir-221-5p	2.91	up

The result of miRNAs chip analysis was tested and verified by applying real-time RT-PCR, the group division was made as previously described, the result of real-time RT-PCR detection was consistent with that of miRNAs chip analysis. Compared with normal CMECs, mir-223-3p up-regulated significantly during the ischemic CMECs proliferation (*P*<0.05) and migration (*P*<0.01) and down-regulated significantly during the tube formation (*P*>0.05) (Figure 5C).

### Rps6kb1 was identified as the direct and functional target of mir-223-3p

**Figure 6:**
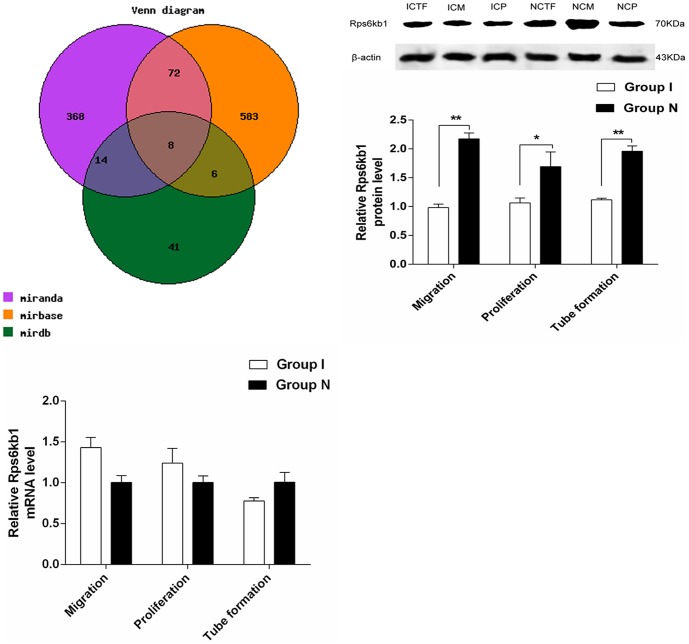
Rps6kb1 was determined as the direct and functional target of mir-223-3p. A. The Venn diagram displayed the overlap between three databases. B. Western blot analyzed the protein level of Rps6kb1 in ICM, ICP and ICTF when compared to that of NCM, NCP and NCTF. β-actin was used as internal controls. (C)Real-time PCR analysis showed no significant difference in RPS6KB1 mRNA expression in ICM, ICP and ICTF when compared to that of NCM, NCP and NCTF. *P<0.05, **P<0.01.

MiRNAs functioned by regulating the expression of target genes [27], two-step sequential approach was used to analyze and confirm the direct and functional target of mir-223-3p: (I) three databases of the target gene of mir-223-3p –mirbase, miranda and mirdb were retrieved to predict the target genes possibly related to the angiogenesis. The genes predicted simultaneously in these three databases were chosen as potential target genes to decrease false positive rate (Fig. 6A), 8 genes were filtered: Acsl3, Alcam, Asz1, Cbfb, Cdk2, Ddit4, Rhoj and Rps6kb1. Next, these genes were analyzed by using pathway, during which Rps6kb1 was found to regulate angiogenesis by participating in HIF-1signal pathway, therefore, it was predicted that Rps6kb1 (Gene ID: 83840) might be the target gene of mir-223-3p.(II) Western blot was applied to detect the protein expression of Rps6kb1, compared with normal CMECs, the protein expression of Rps6kb1 of ischemic CMECs decreased significantly (*P*<0.01) during proliferation and migration, and there was so significant change in tube formation. The mRNA expression of Rps6kb1 was detected by using real-time PCR in order to clarify the reasons of protein expression decreasing: compared with normal CMECs, the mRNA expression of Rps6kb1 of ischemic CMECs had no significant difference, which proved that down-regulation of Rps6kb1 of ischemic CMECs occurred posttransriptionally, the segregation phenomenon of the gene and protein expression of Rps6kb1 conformed to the regulating laws of miRNA, suggesting that Rps6kb1 might be the direct and functional target of mir-223-3p.

mir-223-3p UGUCAGUUUGUCAAAUACCCC

Rps6kb1 241 C**AACTGAC**TG…… 301 GCCGTA**AACTGAC**AGTATTA

### Mir-223-3p inhibiting the angiogenesis of ischemic CMECs by affecting RPS6KB1/hif-1a signal pathway

**Figure 7:**
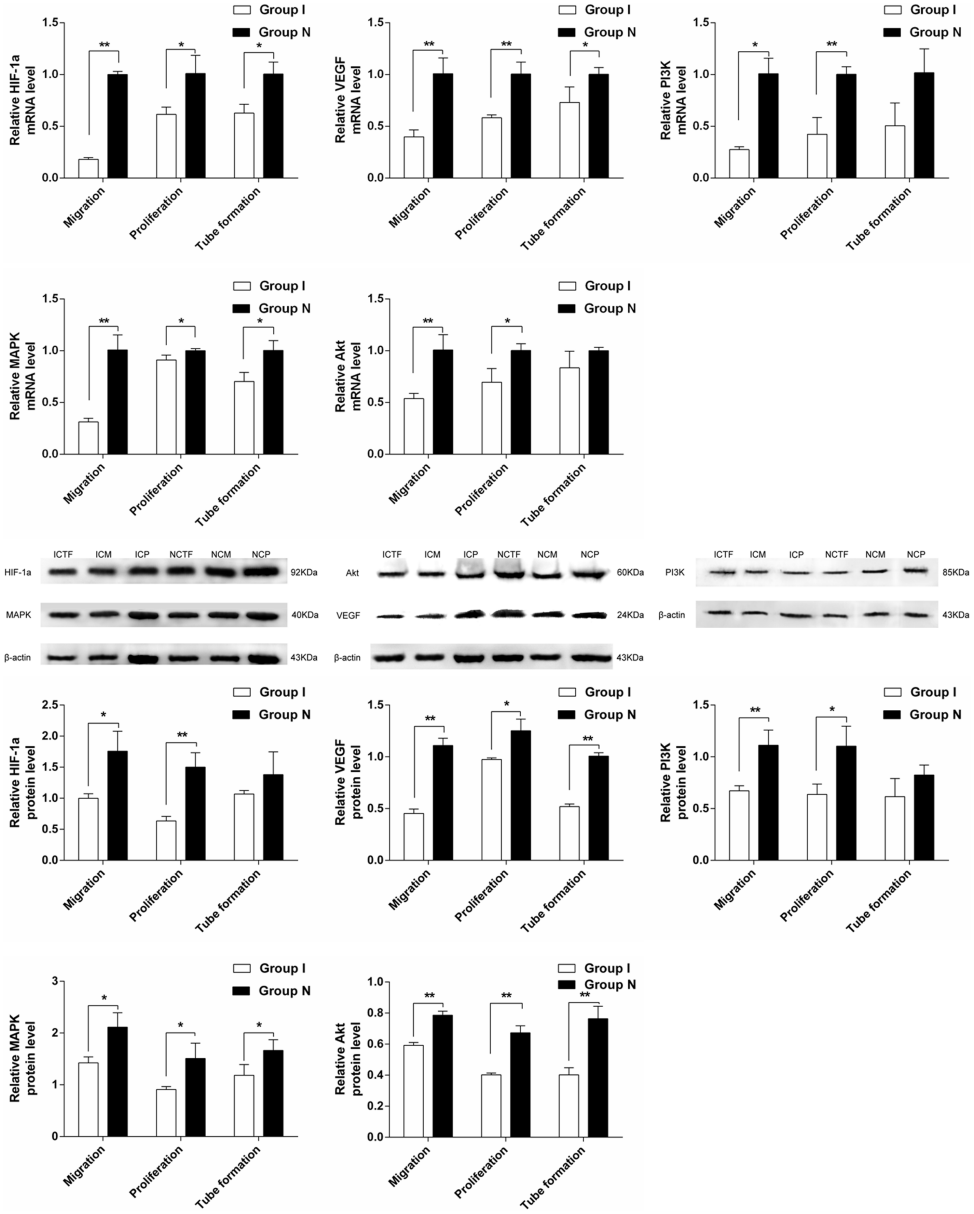
Mir-223-3p inhibited proliferation and migration via affecting RPS6KB1/hif-1a signal pathway. A. Real-time PCR analyzed the mRNA level of hif-1a, VEGF, MAPK, PI3K, Akt in ICM, ICP and ICTF when compared to that of NCM, NCP and NCTF. B. Western blot analyzes the protein level of hif-1a, VEGF, MAPK, PI3K, Akt in ICM, ICP and ICTF when compared to that of NCM, NCP and NCTF. β-actin was used as internal controls. *P<0.05, **P<0.01.

More and more studies had found that many growth factors caused by ischemia and hypoxia functioned via Hif-1a signal pathway [28]–[29]. According to the results of miRNAs chip analysis, it was concluded that Rps6kb1 regulated the angiogenesis by taking part in hif-1a signal pathway. To study the mechanism of mir-223-3p's regulating the angiogenesis of ischemic CMECs; RPS6KB1/hif-1a signal pathway was used as a research target to analyze whether mir-223-3p regulated the angiogenesis via RPS6KB1/hif-1a signal pathway or not. The gene and protein expression of major molecules hif-1a, VEGF, MAPK, PI3K and Akt of RPS6KB1/hif-1a signal pathway were detected by applying real-time PCR and Western bolt, compared with normal CMECs, the gene and protein expression of above mentioned molecules of ischemic CMECs decreased significantly (Figure 7) during proliferation and migration, and the difference in tube formation had no statistical significance, which proved that mir-223-3p decreased the expression of VEGF via RPS6KB1/hif-1a signal pathway, inhibited MAPK, PI3K and Akt in addition to affecting the proliferation and migration of cells, and finally decreased the angiogenesis.

## Discussion

The angiogenic disorders such as decreased myocardial microvascular density and reduced collateral circulation formation etc. are the main pathological changes of ischemic heart diseases. The angiogenesis of microvascular matrix involved the degradation of extracellular matrix, the activation, proliferation, migration, and extension and cell reconstruction of vascular endothelial cells [30]–[31]. Vascular endothelial cells generally existed statistically and transformed into blood capillaries [32] only after the appropriate stimulation. When ischemic cardiovascular diseases attacked, the angiogenesis ability of vascular endothelial cells was limited; however, the characteristics of the angiogenesis of ischemic CMECs, the core miRNA of the angiogenesis of ischemic CMECs and its regulating laws were not clear up till now.

This study found the window phases of the angiogenesis of normal/ischemia CMECs in the process of proliferation, migration, and tube formation (Table 1) by establishing the dynamic model of angiogenesis. It was found that compared with normal CMECs, the proliferation period of ischemic CMECs was significantly delayed, the proliferation ratio and the migration ratio of the ischemic CMECs decreased significantly. In order to further explore the regulatory mechanism of the ischemic CMECs angiogenesis, this study divided them into groups according to the window phases of the proliferation, migration and tube formation in the progress in the normal/ischemic CMECs angiogenesis, miRNA chip hybridization was applied to analyze the expressional changes in the process of the ischemic CMECs angiogenesis and to filter the differentially expressed miRNAs, consequently, there were significant changes of the miRNA gene expression profiles at three different stages of angiogenesis: mir-142-5p up-regulated significantly in ischemic CMECs proliferation, mir-223-3p up-regulated significantly in ischemic CMECs migration and mir-221-5p up-regulated significantly in ischemic CMECs tube formation and, among which mir-223-3p up-regulated most significantly, increased 11.02 times in the migration, and increased 3.68 times in proliferation period, which indicated mir-223-3p played an important role in the process of ischemic CMECs angiogenesis. The mir-223-3p results which were verified via real-time PCR were consistent with those of the chip analysis. Bioinformatics predicted that Rps6kb1 was the potential target gene of mir-223-3p, interestingly, segregation phenomenon of Rps6kb1 mRNA and protein expression was found in the in mir-223-3p- increased-significantly group in the research focused on the expression of Rps6kb1, the segregation phenomenon was consistent with the expression of miRNA regulating gene, which indirect proved that Rps6kb1 probably was the functional target of mir-223-3p.

More and more studies had found that many growth factors caused by ischemia and hypoxia functioned via Hif-1a signal pathway. Pathway analysis indicated that Rps6kb1 regulated the angiogenesis by taking part in hif-1a signal pathway, this complex network contained lots of important molecules, among which mir-223-3p was a critical member. Further analysis revealed that the gene and protein expression of lots of important molecules such as VEGF, MAPK, PI3K and Akt of RPS6KB1/hif-1a signal pathway in the ischemic CMECs decreased significantly during the course of the proliferation and migration, and the difference in tube formation had no statistical significance, which suggested that mir-223-3p decreased the expression of VEGF via RPS6KB1/hif-1a signal pathway, inhibited MAPK, PI3K and Akt in addition to affecting the proliferation and migration of cells ischemic CMECs, and finally decreased the angiogenesis.

Shi L [33] indicate that miR-223 is an antiangiogenic microRNA that prevents endothelial cell proliferation at least partly by targeting β1 integrin. The over-expression of precursor-miR-223 did not affect basal endothelial cell proliferation but abrogated vascular endothelial cell growth factor-induced and basic fibroblast growth factor-induced proliferation, as well as migration and sprouting. MiR-223 over-expression had no effect on the growth factor-induced activation of ERK1/2 but inhibited the vascular endothelial cell growth factor-induced and basic fibroblast growth factor-induced phosphorylation of their receptors and activation of Akt. β1 integrin was identified as a target of miR-223 and its down-regulation reproduced the defects in growth factor receptor phosphorylation and Akt signaling seen after miR-223 overexpression. Reintroduction of β1 integrin into miR-223-ovexpressing cells was sufficient to rescue growth factor signaling and angiogenesis.

Although we found Rps6kb1 could be taken as the potential functional target of mir-223-3p, Rps6kb1 was not the only target gene of mir-223-3p, the function of mir-223-3p came into true through regulating a set of target genes expression after transcription, so we should go on delving into other target genes of mir-223-3p in the future, and to get further comprehensive understanding of mir-223-3p in ischemic CMECs of the progress of angiogenesis. Otherwise, we selected many differentially expressed miRNAs by miRNA chip, mir-223-3p was the most significant of them, and there were other differentially expressed miRNA in ischemic CMECs which played a role in the progress of angiogenesis which needed further research. And then the present study just observed the expressed significantly difference of mir-223-3p in the angiogenesis of the ischemic CMECs, however, whether the intervention of mir-223-3p would improve ischemic CMECs angiogenesis or not remained to be confirmed by further experiments, and we would regulate the expression of mir-223-3p in the following experiments and observe the influence on the angiogenesis of the ischemic CMECs.

In conclusion, this study elucidated the miRNA regulating law in the angiogenesis of CMECs; mir-223-3p inhibited the process of migration and proliferation of ischemic CMECs via affecting RPS6KB1/hif-1a signal pathway, which in turn suppressed the angiogenesis. It is highly possible that mir-223-3p became a novel intervention core target in the treatment of angiogenesis of ischemic heart diseases.
